# Supplementary material for: Association of Lactase Persistence Genotypes (rs4988235) and Ethnicity with Dairy Intake in a Healthy U.S. Population
Source: Nutrients. 2019 Aug 10;11(8):1860. doi: 10.3390/nu11081860 (PMC6723957; doi:10.3390/nu11081860)
Supplement: Supplementary file 1 [file nutrients-11-01860-s001.pdf]

Supplemental Material for

Association of lactase persistence genetics (rs4988235) and ethnicity with dairy intake in a healthy U.S. population

Figure S1. FFQ QC and Raw Data Comparison

Figure S2. ASA24 QC and Raw Data Comparison

Figure S3. Correlations (Kendall) for All Subjects

Figure S4. Correlations (Kendall) for Consumers Only

Table S1. Summary of how fluid milk and alternative milk variables were generated for the FFQ data.

Table S2. Summary of how fluid milk and alternative milk variables were generated for the ASA24 data.

Table S3. Mean (SD) of reported servings and servings/1000-kcal for consumers of each product for the FFQ and ASA24 by genotype.

Table S4. Mean (SD) of reported servings and servings/1000-kcal for consumers of each product for the FFQ and ASA24 by ethnicity.

Supplemental ASA24 QC Methods: Details regarding data quality control for the ASA24 dietary recall data.

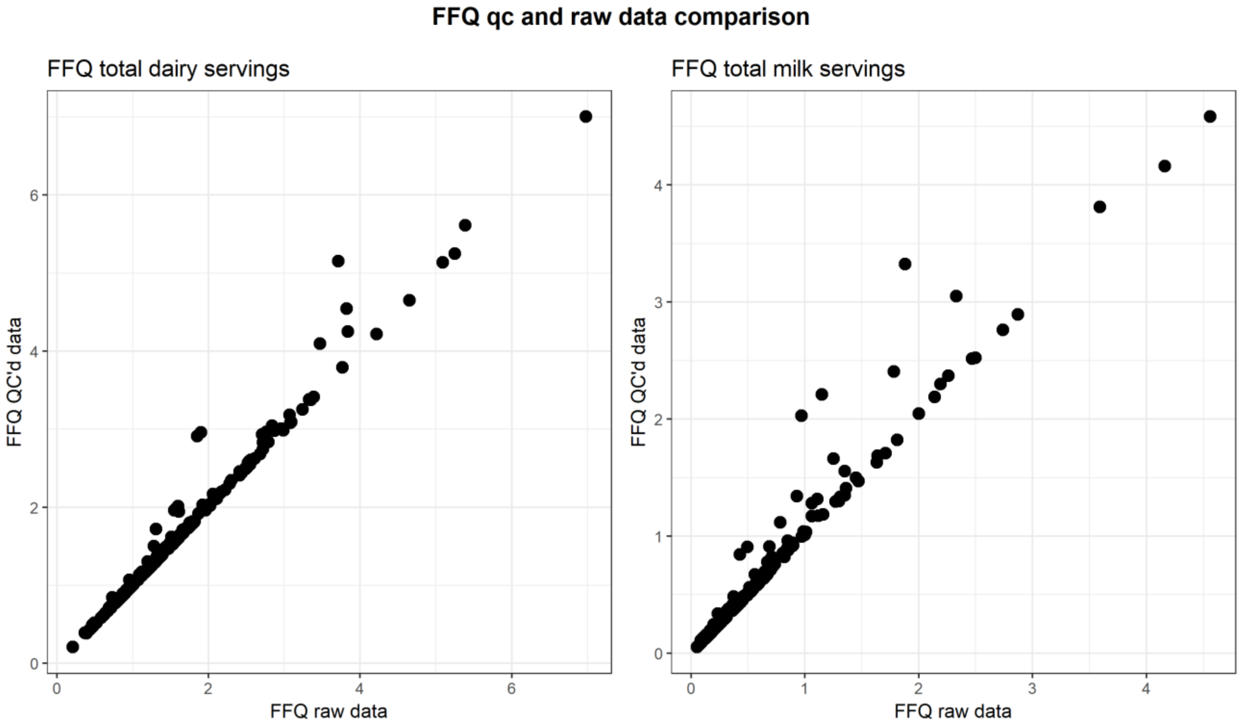

Figure 1. Comparison of FFQ raw and QC'd data. Only the Total Dairy and Milk servings variables were altered due to the inclusion of servings of Soy Milk in both variables. Each marker represents a single questionnaire.

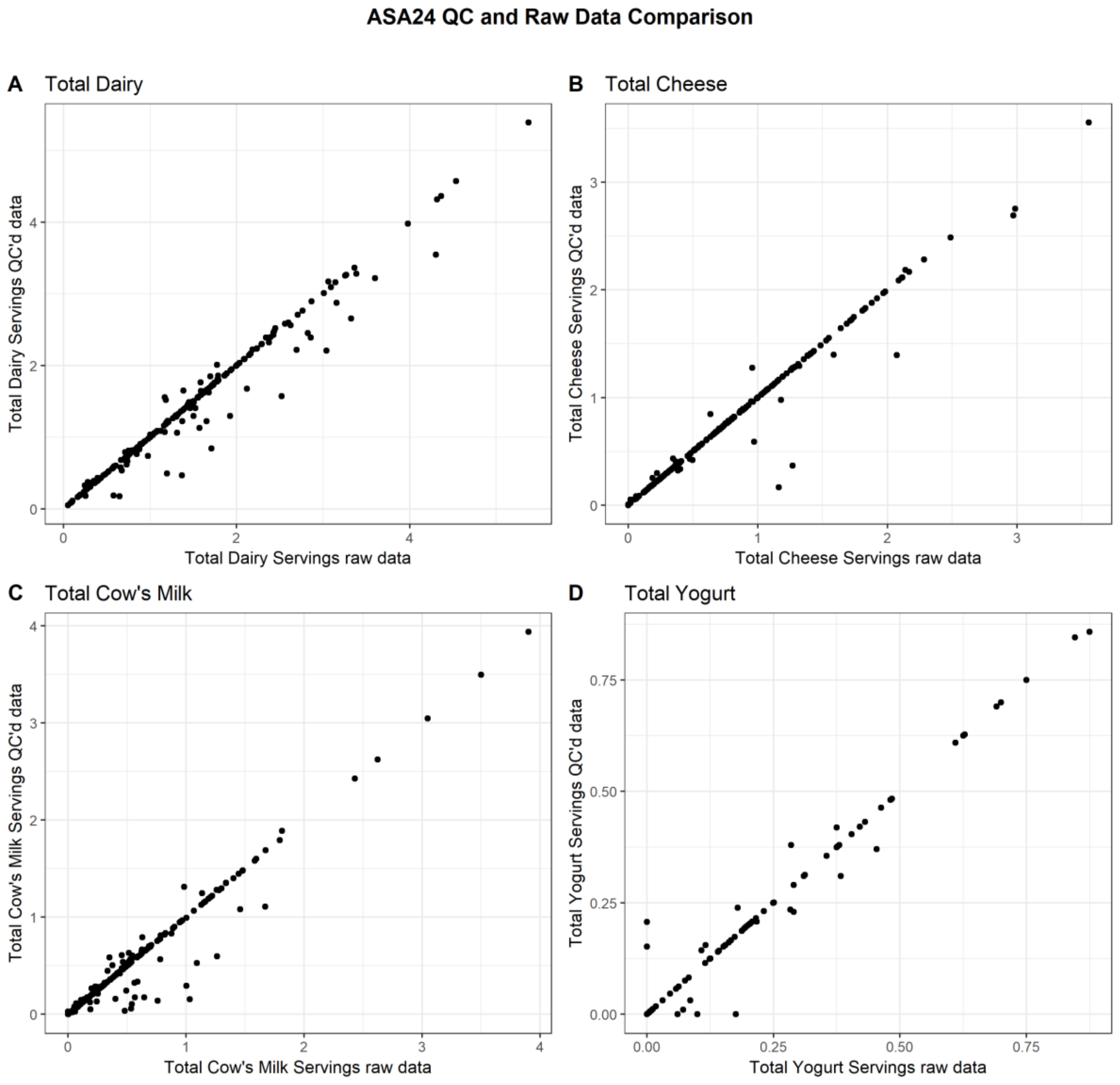

Figure 2. Comparison of QC'd and raw ASA24 Total Dairy (A), Cheese (B), Milk (C), and Yogurt (D). Each marker represents a single recall.

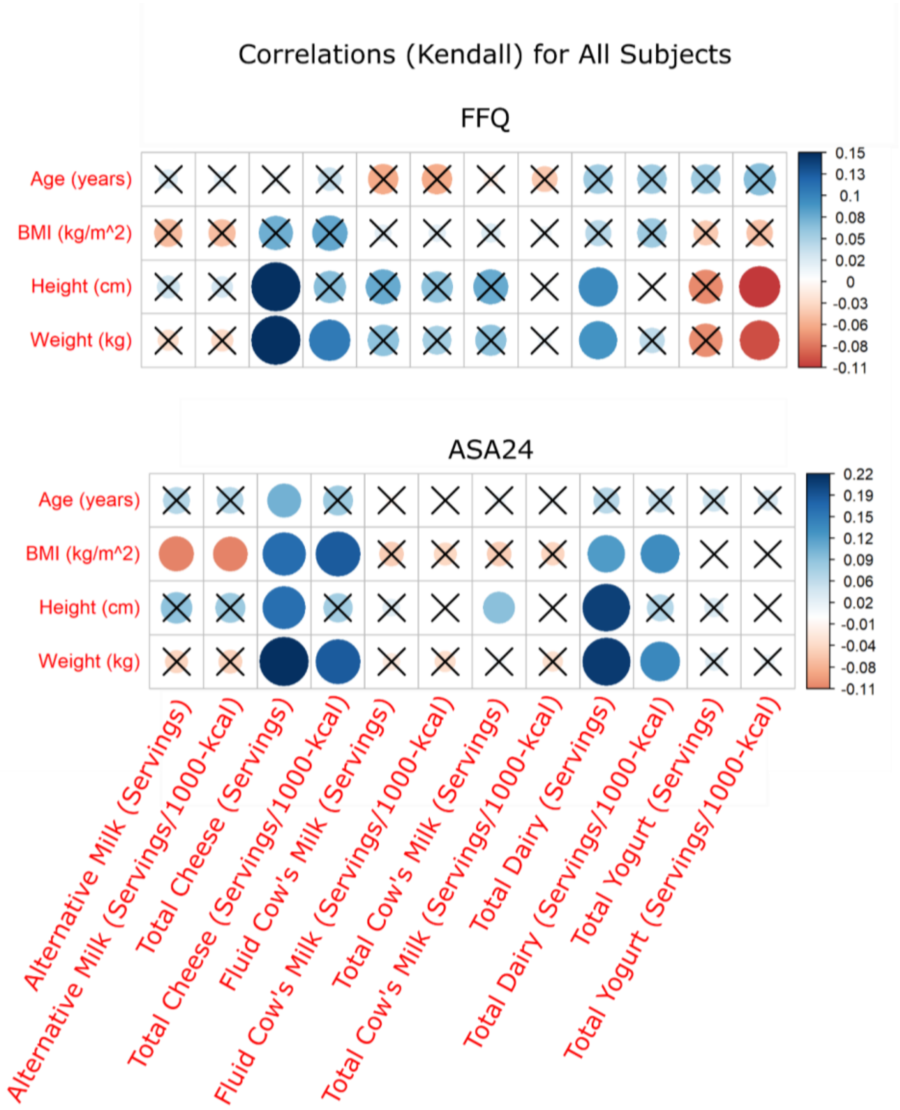

Figure 3. Correlation plots between age, BMI, height, and weight and the FFQ (n = 218 (top)) and ASA24 (n = 214 (bottom)) for both consumers and non-consumers of products (Kendalls' τ). A larger marker and darker blue color indicate a larger positive correlation; a larger marker and brighter orange color indicate a larger negative correlation. An X indicates no significant correlation between dairy variables and metadata ($p > 0.05$).

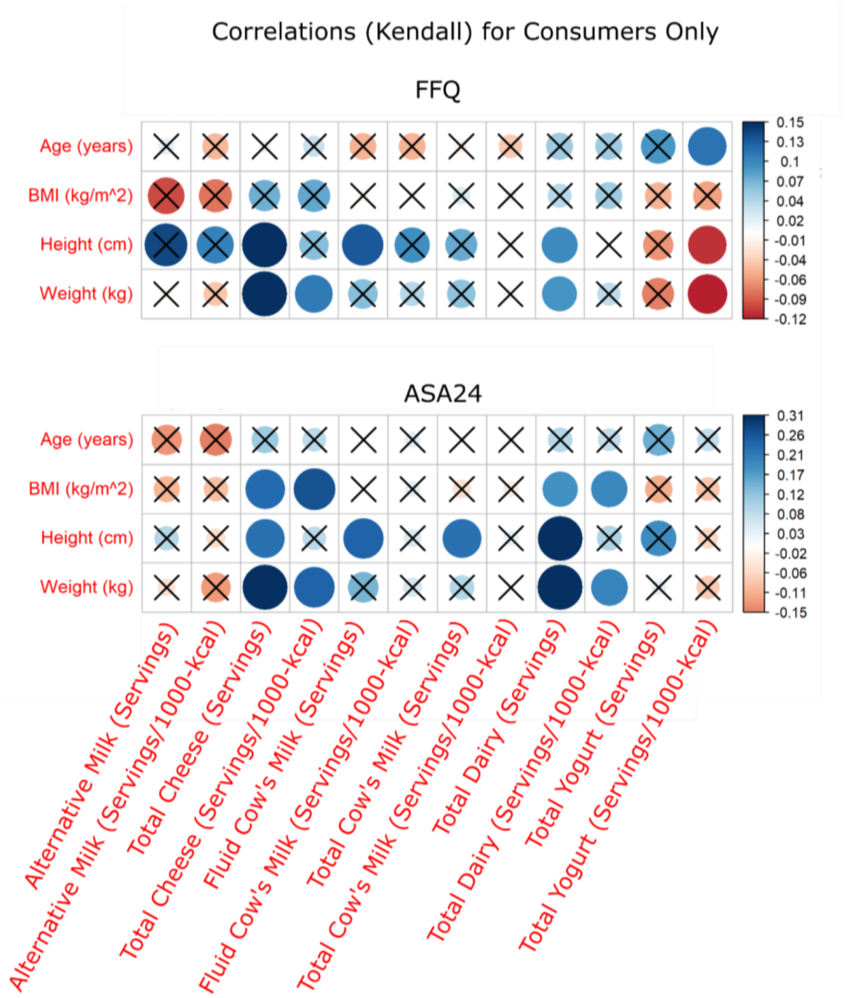

Figure 4. Correlation plots between age, BMI, height, and weight and the FFQ (n = 218 (top)) and ASA24 (n = 214 (bottom)) for only the consumers of each product (Kendalls' τ). For both ASA24 and FFQ, a consumer of total dairy, total cheese, fluid cow's milk, and alternative milk reported > 0 servings and ≥ 0.0333 servings of yogurt; a total cow's milk consumer reported > 0.1 servings for the ASA24 and > 0 servings for the FFQ; A larger marker and darker blue color indicate a larger positive correlation; a larger marker and brighter orange color indicate a larger negative correlation. An X indicates no significant correlation between dairy variables and metadata (p > 0.05).

Table 1. Summary of how fluid milk and alternative milk variables were generated for the FFQ data.

**MILKTYPE response[1]**	**Assigned to…**	**Subtracted from D_TOTAL (Total Dairy) and D_MILK (Total Milk)?**
Whole, 2%, 1%, skim, or Don't Drink	Fluid Cow's Milk	No
Soy	Alternative Milk	Yes
Almond or Rice milk	Alternative Milk	No

[1]The Block FFQ default is to assign any reported milk as 2% if the subject's response to MILKTYPE is "Don't Drink.".

Table 2. Summary of how fluid milk and alternative milk variables were generated for the ASA24 data.

**Food Description Keywords (FoodCode)**	**Assigned to…**	**Subtracted from D_TOTAL (Total Dairy) and D_MILK (Total Milk)?**
Whole (11111000)<br>2% (11112110)<br>1% (11112210)<br>Skim (11113000)<br>Milk, NFS (11100000)<br>Chocolate, skim[1] (11511300)<br>Lactose reduced, 2%[1] (11114330)	Fluid Cow's Milk	No
Soy Milk (11320000)	Alternative Milk	Yes
Almond Milk (11350000)<br>Rice Beverage (92205000)	Alternative Milk	No

[1] No other types were reported in the recalls (i.e. whole, 1% etc.).

Table 3. Mean (SD) of reported servings and servings/1000-kcal for consumers of each product for the FFQ and ASA24 by genotype.

	**Habitual Intake (FFQ)**					**Recent Intake (ASA24)**				
	**AA**	**AG**	**GG**	**p[1]**	**p[2]**	**AA**	**AG**	**GG**	**p[1]**	**p[2]**
					**Servings·day**					
Total Dairy[3]	1.86 (1.06)	1.68 (1.07)	1.62 (1.01)	0.279	0.196	1.57 (1)[ab]	1.64 (1)[a]	1.25 (0.76)[b]	0.019	0.005
Total Cheese[3]	0.87 (0.5)	0.8 (0.47)	0.74 (0.54)	0.105	0.043	0.83 (0.59)	0.93 (0.68)	0.73 (0.55)	0.179	0.076
Total Yogurt[4]	0.23 (0.21)	0.24 (0.18)	0.28 (0.32)	0.649	0.548	0.34 (0.2)	0.27 (0.16)	0.31 (0.23)	0.324	0.445
Total Milk[3]	0.74 (0.84)	0.67 (0.75)	0.62 (0.69)	0.632	0.402	0.66 (0.79)	0.69 (0.53)	0.54 (0.51)	0.181	0.124
Fluid Milk[4]	0.51 (0.8)	0.57 (0.76)	0.45 (0.58)	0.574	0.722	0.7 (0.94)	0.57 (0.45)	0.56 (0.57)	0.715	0.669
Alt. Milk[4]	0.3 (0.31)	0.41 (0.75)	0.35 (0.46)	0.962	0.798	0.33 (0.3)	0.91 (1.02)	0.55 (0.43)	0.056	0.949
					**Servings/1000-kcal·day**					
Total Dairy[3]	0.93 (0.48)	0.85 (0.38)	0.81 (0.37)	0.297	0.250	0.73 (0.42)	0.74 (0.37)	0.62 (0.35)	0.075	0.023
Total Cheese[3]	0.44 (0.23)	0.41 (0.2)	0.37 (0.22)	0.114	0.051	0.39 (0.26)	0.42 (0.29)	0.36 (0.23)	0.440	0.220
Total Yogurt[4]	0.12 (0.1)	0.13 (0.11)	0.16 (0.23)	0.707	0.477	0.16 (0.09)	0.12 (0.06)	0.15 (0.11)	0.355	0.790
Total Milk[3]	0.37 (0.42)	0.33 (0.31)	0.3 (0.3)	0.715	0.496	0.3 (0.33)	0.31 (0.24)	0.28 (0.26)	0.414	0.267
Fluid Milk[4]	0.26 (0.45)	0.26 (0.3)	0.21 (0.24)	0.490	0.734	0.32 (0.4)	0.26 (0.17)	0.29 (0.3)	0.718	0.893
Alt. Milk[4]	0.15 (0.18)	0.18 (0.32)	0.18 (0.22)	0.635	0.352	0.15 (0.13)	0.38 (0.38)	0.28 (0.29)	0.065	0.806

Within a genotype group, means without a common superscript letter differ ($p < 0.05$). Mean (SD) are for servings or servings/1000 kcal, but p values are from models using transformed data. [1] p value for model with all rs4988235 genotypes as the predictor. [2] p value for model with LP genotype as the predictor (LP = AA and AG; LNP = GG). [3] from linear model with no covariates. [4] from ordinal logistic regression with no covariates (adjusted models had similar p values.

Table 4. Mean (SD) of reported servings and servings/1000-kcal for consumers of each product for the FFQ and ASA24 by ethnicity.

	**Habitual Intake (FFQ)**						**Recent Intake (ASA24)**					
	**African American**	**Asian**	**Caucasian**	**Hispanic**	**p[1]**	**p[2]**	**African American**	**Asian**	**Caucasian**	**Hispanic**	**p[1]**	**p[2]**
**Servings·day**												
Total Dairy[3]	1.41 (0.41)	1.43 (1.08)	1.72 (1.06)	1.68 (0.87)	0.242	0.226	1.22 (0.91)[ab]	1.01 (0.62)[a]	1.6 (0.98)[b]	1.35 (0.76)[ab]	0.014	0.005
Total Cheese[3]	0.91 (0.42)[a]	0.53 (0.5)[b]	0.82 (0.47)[a]	0.79 (0.49)[a]	0.002	0.036	0.89 (0.67)[ab]	0.46 (0.4)[a]	0.88 (0.6)[b]	0.78 (0.6)[ab]	0.005	0.015
Total Yogurt[4]	0.11 (0.06)	0.24 (0.24)	0.24 (0.19)	0.25 (0.23)	0.655	0.455	0.22 (0.02)	0.33 (0.2)	0.31 (0.2)	0.3 (0.25)	0.751	0.647
Total Milk[3]	0.43 (0.21)	0.63 (0.75)	0.67 (0.79)	0.64 (0.53)	0.785	0.805	0.52 (0.45)	0.58 (0.45)	0.68 (0.66)	0.53 (0.61)	0.499	0.126
Fluid Milk[4]	0.2 (0.17)	0.58 (0.74)	0.52 (0.79)	0.47 (0.5)	0.557	0.555	0.78 (0.47)	0.51 (0.42)	0.62 (0.68)	0.69 (0.89)	0.728	0.867
Alt. Milk[4]	0.33 (0.31)	0.57 (0.74)	0.37 (0.61)	0.33 (0.47)	0.899	0.638	0.41 (0.23)	0.61 (0.28)	0.65 (0.88)	0.68 (0.48)	0.553	0.194
**Servings/1000-kcal·day**												
Total Dairy[3]	0.69 (0.28)	0.73 (0.27)	0.9 (0.44)	0.78 (0.24)	0.153	0.040	0.58 (0.45)	0.53 (0.31)	0.74 (0.38)	0.66 (0.36)	0.044	0.015
Total Cheese[3]	0.45 (0.25)[a]	0.27 (0.18)[b]	0.43 (0.23)[a]	0.36 (0.11)[ab]	0.000	0.004	0.42 (0.29)[ab]	0.25 (0.19)[a]	0.41 (0.27)[b]	0.36 (0.21)[ab]	0.029	0.030
Total Yogurt[4]	0.05 (0.03)	0.13 (0.14)	0.13 (0.1)	0.12 (0.11)	0.206	0.292	0.08 (0.01)	0.19 (0.12)	0.14 (0.09)	0.15 (0.13)	0.448	0.920
Total Milk[3]	0.21 (0.14)	0.32 (0.27)	0.34 (0.37)	0.3 (0.23)	0.741	0.961	0.25 (0.21)	0.29 (0.24)	0.3 (0.27)	0.3 (0.36)	0.665	0.291
Fluid Milk[4]	0.1 (0.11)	0.25 (0.25)	0.26 (0.38)	0.23 (0.24)	0.520	0.736	0.34 (0.2)	0.26 (0.21)	0.28 (0.29)	0.4 (0.46)	0.872	0.713
Alt. Milk[4]	0.11 (0.07)	0.25 (0.2)	0.18 (0.27)	0.21 (0.33)	0.575	0.494	0.17 (0.11)	0.35 (0.2)	0.27 (0.33)	0.38 (0.38)	0.404	0.159

Within a group, means without a common superscript letter differ. Means (SD) for servings or servings/1000 kcal, but *p* values are from models using transformed data. [1] *p* value for model with Ethnicity as the predictor. [2] *p* value for model with whether subjects identified as Caucasian or not as the predictor. [3] from linear model with no covariates. [4] from ordinal logistic regression with no covariates (adjusted models had similar *p* values).

Supplemental ASA24 QC Methods

FoodCodes, food descriptions, food amounts (g), and the calculated output from the system for servings of dairy (total dairy: D_TOTAL; total cheese: D_CHEESE; total yogurt: D_YOGURT; and total milk: D_MILK) were retrieved from the *INFMYPHEI* and *Items* files for ASA24-2014 and 2016, respectively. The total amount of calories consumed per subject per recall was retrieved from the *TNMYPHEI* and *Totals* files for ASA24-2014 and 2016, respectively. Data by respondent from the probe questions were reviewed in files *MS* and *Responses* for ASA24-2014 and 2016, respectively. The *INS* file was used to identify subjects who consumed the lactase enzyme for both ASA24-2014 and 2016; no subjects recorded use of the lactase enzyme.

In both ASA24-2014 and ASA24-2016, soy milk (FoodCode: 11320000) contributed to the D_MILK variable (and therefore total dairy (D_TOTAL)); any soy milk contribution to D_MILK was subtracted from D_MILK and total dairy (D_TOTAL), then added to the "alternative milk" variable (D_ALT) that we created for this study. ASA24-2016 had a unique FoodCode for "almond milk" (FoodCode: 11350000), but no almond milk FoodCode existed in ASA24-2014. In ASA24-2014, subjects consuming almond milk typically selected "Milk, NFS" (FoodCode: 11100000). and then wrote in "almond milk" in their response file. We therefore replaced any responses of "Milk, NFS" with a write-in response of "almond" or "almond milk" with the FoodCode 11350000, subtracted the servings from D_MILK and D_TOTAL, and added the servings to D_ALT. Additionally, D_MILK servings for write-in foods such as soy ice cream, latte with almond milk and latte with soy milk, was subtracted from D_MILK and D_TOTAL, and the servings was added to D_ALT. Servings corresponding to write-in responses relating to "kefir" were added to the total yogurt variable (D_YOGURT).

A variable corresponding to servings of fluid cow's milk consumed as a beverage, with cereal, or in coffee/tea (i.e. not from a mixed recipe) was generated. First, we searched response files for food descriptions containing the word "milk". The values for the resulting food codes corresponding to Whole Milk, (11111000), 2% Milk (11112110), 1% Milk (11112210), Skim Milk (11113000), Milk, NFS (11100000), Chocolate skim milk (11511300), and 2% Lactose-reduced milk (11114330) were added to the fluid cow's milk variable if the subject did not write in any alternative milk description (**Table S2**).

ASA24-2014 relies on the USDA Food and Nutrient Database for Dietary Studies (FNDDS) 4.1 (2007/2008) while ASA24-2016 relies on FNDDS 2011-2012. There were discrepancies in the way that some cow's milk products contributed to the total milk (D_MILK) and total dairy (D_TOTAL) variables between ASA24-2014 and ASA24-2016. In ASA24-2014, sour cream (12310100), sour cream, lite (12310350), sour cream, reduced fat (12310300), sour cream, fat free (12310370), cream, NS (12100100), cream, half and half (12120100), cheese, cream (14301010), cream cheese, light (14303010), and cream cheese, regular (14420200) did not contribute to any dairy, but did in ASA24-2016. The Food Pattern Equivalents Database cup equivalent weights [1] were used to convert the grams of each food to cup equivalents (servings). A two tailed Kolmogorov-Smirnov test and a t-test revealed no significant differences in the distribution or the means, respectively, of dairy variables after data QC ($p > 0.05$).

Many subjects reporting consuming low amounts of cow's milk in ASA24 recalls; further inspection of subjects' response files revealed that these low amounts were from mixed recipes where small amounts of milk were added. The amount of milk from mixed recipes was estimated by subtracting the servings of fluid milk from D_MILK. The average amount of milk from mixed recipes was 0.1 servings, which was used to define a reported 'consumer' of milk for ASA24 analyses.

A recall was excluded from analysis if it was incomplete or if the total kcal was not within the NHANES 5th to 95th percentiles. A recall was defined as incomplete if subjects failed to report details for five or more foods consecutively per recall; this made nutrient calculations unreliable. Following these steps, subjects with a minimum of three recalls were included in analysis (n = 214).

References

1. Bowman, S.A.; Clemens, J.C.; Friday, J.E.; Thoerig, R.C.; Shimizu, M.; Barrows, B.R.; Moshfegh, A.J. Food Patterns Equivalents Database 2007-08: Methodology and User Guide Available online: https://www.ars.usda.gov/ARSUserFiles/80400530/pdf/fped/FPED_1516.pdf (accessed on Mar 22, 2019).
